# Doctrines of the Circulation

**Published:** 1885-01

**Authors:** 


**Doctrines of the Circulation.** A History of Physiological Opinion and Discovery in Regard to the Circulation of the Blood. By J. C. DALTON, M. D., Professor Emeritus of Physiology in the College of Physicians and Surgeons, New York, and President of the College. Philadelphia: Henry C. Lea's Sons & Co. 1884.

Professor Dalton gives, in this work, a history of physiological opinion and discovery as to the circulation of the blood from the times and writings of Aristotle down to the present century. It is not a work for criticism, being a historical as well as scientific presentation of this subject, which the author gives in his usual clear and common style. To students in physiology who desire to trace the theories of the circulation of the blood to their source, and investigate the gradual solution of this difficult problem, we especially commend the work.

